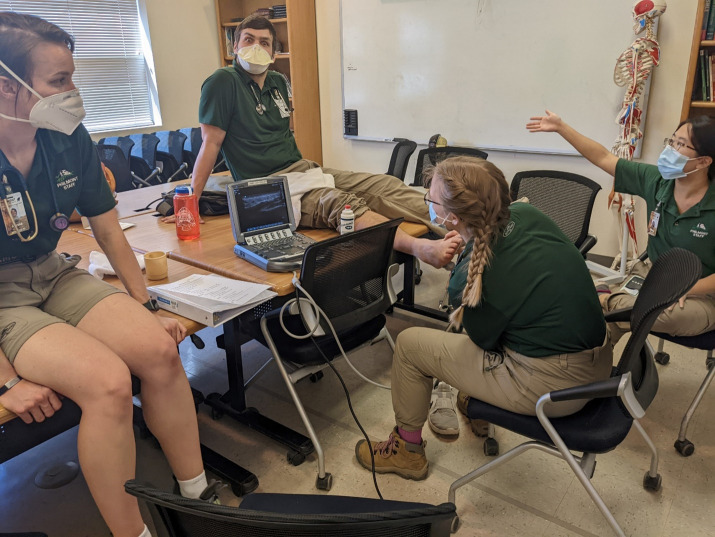# Piloting a Diagnostic Foot and Ankle Fracture Sonographic Algorithm with Rural and Adolescent Patients

**DOI:** 10.24908/pocus.v9i2.17550

**Published:** 2024-11-15

**Authors:** Tomas Alamin, Margaret Lin­-Martore, Aaron E Kornblith, Aidan O'Donnell, Sally Gragalia

**Affiliations:** 1 Department of Emergency Medicine, University of California San Francisco, CA USA; 2 Department of Pediatrics, University of California San Francisco, CA USA; 3 Human Biology Division, Fred Hutchinson Cancer Center Seattle, WA USA

**Keywords:** Point-of-Care Ultrasound, POCUS, Ankle, Rural, Resource-Limited Setting, Adolescent, POCUS Training

## Abstract

**Background: **Foot and ankle injuries are a common presenting complaint to the Emergency Department (ED) and are often assessed with plain radiography. Rural environments may not have access to radiography mandating the referral or transfer patients to regional centers for definitive diagnosis. The Ottawa Foot and Ankle Rules (OFAR) is a clinical decision rule that can assist in ruling out fractures. Point of care ultrasound (POCUS) can augment this decision rule. The objective of this study was to assess both the feasibility and test characteristics of a previously described POCUS augmented clinical assessment, OFAR-POCUS, for adolescent and adult patients with foot and ankle pain in a rural environment. **Methods:** This was a prospective cohort study from June to August 2022 including patients with chief complaint of foot or ankle injury presenting to a rural clinic. Patients were included if they had positive finding(s) on the OFAR Test and required radiographic imaging. Patients were excluded if they did not consent, speak English, were unable to be scanned, had obvious joint deformities, had altered mental status, were not physiologically stable, had other injuries preventing sonography, were pregnant, or had previous injury with internal fixation, osteomyelitis, or rheumatoid arthritis. POCUS was performed before transport for radiography. POCUS examiners were POCUS novices who underwent a one and a half to two-hour, standardized foot and ankle POCUS training session. All POCUS studies were reviewed by two emergency medicine ultrasound fellowship trained faculty for quality assurance. Standard test characteristics were calculated for bedside clinician and expert POCUS interpretations compared to the radiographic control. **Results:** Thirteen POCUS examiners performed exams on 20 patients included in analysis; four patients had fractures on radiograph (20%). The bedside clinician POCUS interpretation had sensitivity (SN) = 100% (95% Cl, 40%-100%), specificity (SP) =94% (95% Cl, 70%-100%), and negative likelihood ratio (-LR) = 16.00 (95% Cl, 2.40-106.73). Expert POCUS interpretation had SN=75% (95% Cl, 19%-99%), SP=75% (95% Cl, 48%-93%), and -LR=0.33 (95% Cl, 0.06-1.86). **Conclusion:** A POCUS enhanced clinical strategy for clinically significant foot and ankle fractures in adolescent and adult patients in a rural setting is feasible. Larger studies are required to further characterize test characteristics and use of foot and ankle POCUS where plain radiography is unavailable.

## Introduction

Foot and ankle injuries are a common presenting complaint to the Emergency Department (ED) with an annual incidence of 14.1/10,000 for ankle fractures^1^ and 14.2/10,000 for foot fractures 2. Many patients who present with foot and ankle pain undergo radiographic imaging to receive a definitive diagnosis. However, in many low resource medical settings, radiographic imaging may not be readily available, and can require hours of transportation and additional allocation of already scarce resources. In such locations, patients suffer increased costs, delayed treatment, and decreased satisfaction. Therefore, bedside screening tools for low-likelihood-fracture-patients are useful. Currently, the Ottawa Foot and Ankle Rules (OFAR) fill this role in many clinics without convenient access to radiography. However, the test characteristics of the OFAR has a specificity (SP) of 25%-42% 3, 4, leaving room for improvement. 

Point of care ultrasound (POCUS) has proved to be a cost-effective and rapid diagnostic aid in both high resource and low resource medical environments. POCUS is also helpful in the diagnosis of clinically significant foot and ankle fractures (defined as >3 mm and non-evulsion), especially when combined with the OFAR (OFAR-POCUS). OFAR-POCUS algorithms have a high SP (80%-94%) 5, 6. Unfortunately, most studies using POCUS to augment the OFAR occurred in urban or suburban EDs where radiography is easily accessible, and providers have extensive POCUS training and expertise. Not infrequently, physicians in rural environments do not have as much POCUS experience as urban emergency medicine physicians 7. Therefore, to date, the OFAR-POCUS algorithm has not been tested in the locations where it would be most useful. Previous studies have only studied the OFAR-POCUS algorithm in adults and young pediatric patients (<12 years old). Adolescents, from 12 to 17 years old, still have open growth plates, which can appear similar to fractures on ultrasound. They also have differing musculoskeletal anatomy than younger pediatric patients. Together, these realities necessitate further investigation of the OFAR-POCUS algorithm in adolescent populations.

To address this gap, the OFAR-POCUS algorithm was implemented in a rural clinic location (Cimarron, New Mexico, population 792 8) for both adolescent and adult participants. The objective of this study was to assess both the feasibility and test characteristics of a POCUS augmented clinical assessment adapted from previous studies 5, 6, OFAR-POCUS, for adolescent and adult patients with foot and ankle pain in a rural environment. The hypothesis was that novice POCUS practitioners could learn and use a standardized OFAR-POCUS algorithm after a short training session with high SP and sensitivity (SN).

## Methods

**Figure 1:**
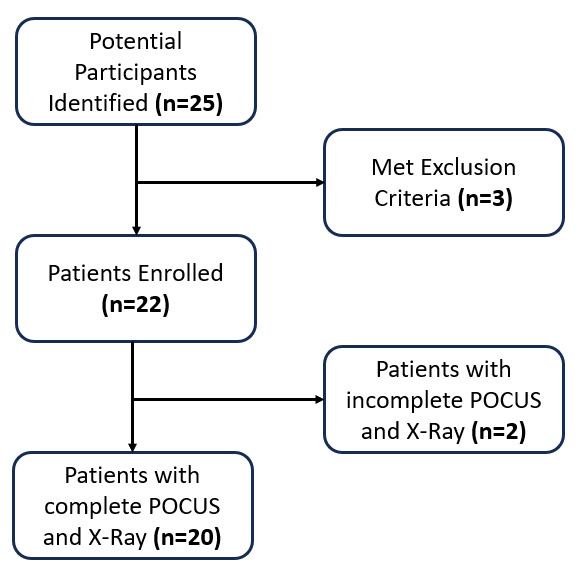
Study population flowchart. Flowchart showing which patients were included or excluded from the study.

This study was a prospective case-control study assessing POCUS’s utility in diagnosing clinically relevant (>3 mm and non-evulsion) foot and ankle fractures. OFAR positive patients were enrolled at the Philmont Infirmary, a small rural medical clinic in Cimarron, New Mexico, affiliated with a Boy Scout high adventure camp, with a 2022 annual patient volume of 3216. The study began in June 2022 and ended in August 2022. The institutional review board approved this study, and all researchers obtained informed consent for all patients. This was a single institution study. The University of California San Francisco Institutional Review Board approved the study (# 22-36381). Study recruitment is shown in Figure 1. 

### Participants

Patients were youth and adults participating in 7-12 day backpacking trips who sustained injuries during outdoor recreation. Participants were derived from a convenience sample of prospectively identified patients presenting to clinic with lower extremity injuries. Participants under 18 years of age provided written assent and legal guardians were contacted to provide written consent for the participation. Participants 18 years and older provided written consent for themselves. Inclusion criteria and exclusion criteria were as follows:

### Inclusion criteria

Patients age 12 and older with self-reported foot or ankle pain being sent for a radiograph of the foot or ankle

### Exclusion criteria

- Patients who cannot communicate in English
- Patients who do not consent to participate
- Patients with open wounds when the provider is not able to find or use a barrier for an ultrasound probe
- Patients with obvious foot or ankle deformities
- Patients with altered mental status
- Trauma patients with acute life-threatening ailments/unstable patients
- Minors for whom study staff are unable to contact parents for verbal consent
- Patients with multiple injuries preventing foot and ankle POCUS before radiography
- Pregnant patients
- Patients with previous injury including internal fixation, osteomyelitis, rheumatoid arthritis

### Procedure

All patients were initially evaluated with a history and physical exam, including the OFAR exam, in accordance with usual care. When patients received a positive OFAR exam, they were approached to join the study. POCUS was conducted after positive OFAR findings and before participants had radiograph imaging of the injured extremity. The study design is shown in Figure 1. During enrollment, two participants who received OFAR-POCUS did not receive radiography and were excluded from the study. Study staff identified these participants by reviewing their electronic medical record (EMR) charts when confirming inclusion status and excluded their data from all calculations and analysis.

All POCUS examiners, with the exception of the first author, underwent a one and a half to two-hour standardized training session on performing foot and ankle skeletal POCUS using the algorithm outlined below. The first author led all training sessions. The instructional POCUS session involved 1) a 45-slide didactic slideshow as well as 2) a hands-on portion where examiners were able to practice POCUS techniques with other examiners, as shown in Appendix 1. All POCUS examiners were fourth year medical students, with the exception of the first author who was a second-year medical student who had received 20 hours of general POCUS instruction and 12 hours of direct instruction from a POCUS-fellowship trained expert on the OFAR-POCUS algorithm. 

**Figure 2:**
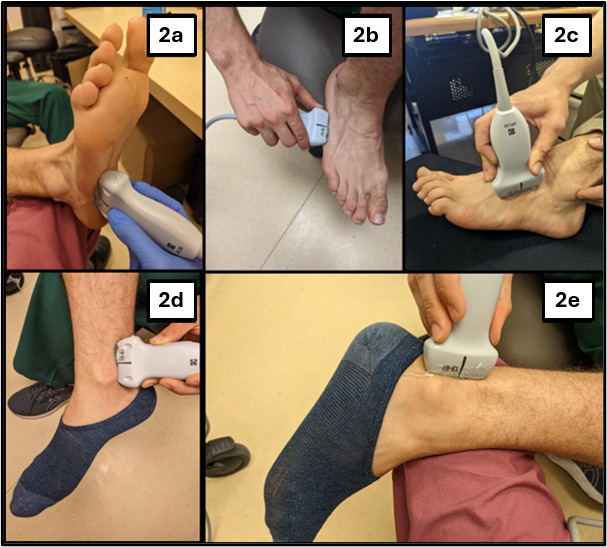
Ottawa Foot and Ankle Rules Point of Care Ultrasound (OFAR-POCUS) algorithm. Above are ultrasound locations assessed in the sonographic algorithm, demonstrating probe placement. Ultrasound probes are depicted at the starting points for sonograph recordings. Figure 2a shows positioning for the inferior 5th metatarsal view. Figure 2b shows positioning for the lateral 5th metatarsal view. Figure 2c shows positioning for the superior 5th metatarsal view. Figure 2d shows positioning for the medial malleolus view.

The POCUS examiners performed POCUS exams of participants’ injured lower extremities. POCUS examiners were usually the same individuals who conducted the patients’ initial history and physical exams. If someone was unavailable or had not received the research-specific POCUS training, another POCUS examiner conducted the POCUS exam. When a different individual was used for the POCUS exam, that POCUS examiner would reconfirm the positive OFAR before beginning POCUS. POCUS examiners used the following OFAR-POCUS sonographic algorithm, also shown in Figure 2.

a. The inferior aspect of the 5th metatarsal from the tarsal-metatarsal joint distally to the metatarsal-phalangeal joint.

b. The lateral aspect of the 5th metatarsal from the tarsal-metatarsal joint distally to the metatarsal-phalangeal joint.

c. The superior aspect of the 5th metatarsal from the tarsal-metatarsal joint distally to the metatarsal-phalangeal joint.

d. The posterior aspect of the tibia from the medial malleolus to 10 cm proximal of the medial malleolus.

e. The posterior aspect of the fibula from the lateral malleolus to 10 cm proximal of the lateral malleolus.

POCUS examiners were allowed to repeat scans during the exam. The 10 cm distances were measured with a ruler or estimated based on examiner preference. POCUS examiners were instructed to overestimate lengths if they did not use the ruler. The study used a 10 cm length instead of the 6 cm length used in the OFAR exam as previous OFAR-POCUS studies observed fractures between 6 cm and 10 cm proximal of the malleoli 5. POCUS examiners also completed a data form with participants which recorded patient age, gender, injury time, time of POCUS exam start, time of POCUS exam conclusion, mechanism of injury, positive OFAR criteria, and POCUS findings.

After POCUS was complete, all participants received radiograph imaging at Miners Colefax Medical Center in Raton (MCMC), New Mexico, as part of usual care. MCMC radiology staff and radiology contractors interpreted the images, and researchers recorded their interpretations from the EMR. MCMC staff were unaware of POCUS results.

After data collection was complete, all POCUS images were sent to two POCUS-fellowship trained Emergency Medicine physicians for review. The reviewers were blinded from all clinical data (history, physical exam findings, OFAR results), primary POCUS impressions, and radiograph impressions. The reviewers marked all experimental sonographs as positive, negative, or indeterminate. Positive indicated that the reviewer thought there was a high certainty of a fracture. Negative indicated the reviewer felt there was no evidence of a fracture. Indeterminate meant that the sonographic images were inconclusive for evidence of a fracture. Afterwards, the reviewers met and came to a consensus on discordant impressions. As inconclusive results reflected the inability to rule out a fracture, they were considered statistically positive findings in all calculations. Finally, the radiographic diagnosis was compared with those of the bedside POCUS examiners and the expert secondary reviewers. 

### Materials

All scans were done using a Sonosite M-Turbo machine or a Butterfly IQ+. The M-Turbo was used for 19 of the scans; the Butterfly IQ+ was used for three. Examiners recorded exams via sonographic clips for retrospective analysis by POCUS experts. Clip length was set to six seconds on the Sonosite M-Turbo and one minute on the Butterfly IQ+. A linear probe was always used with the M-Turbo in the “Musculoskeletal” (MSK) mode. “Fascial-Vascular” and “Ophthalmologic” were the primary IQ+ modes used. 

### Outcome Measures

Clinically irrelevant fractures were considered negative for this experiment. If a fracture was an avulsion fracture smaller than 3 mm in length, it was considered insignificant, in accordance with prior studies 5, 6. Salter-Harris 1 fractures were considered significant, though their clinical course and treatment is more similar to that of a sprain. 

### Statistical Methods

For all statistics, radiographic diagnosis served as diagnostic control. Sonographic results served as test variables. Using these inputs, SN, SP, positive predictive value (PPV), negative predictive value (NPV), positive likelihood ratio (+LR), and negative likelihood ratio (-LR) were calculated in the standard fashion using the statistical software R 9. These values were calculated for both the bedside and expert diagnosis, yielding two sets of values. Bedside examiners and expert reviewers were allowed to select between “fracture,” “no fracture,” and “indeterminate” when judging sonographs. Bedside examiners selected indeterminate when they did not see evidence of fracture but simultaneously were not able to visualize cortex adequately due to artifact, technique limitation, or other view-obscuring phenomena. Indeterminate diagnoses were considered fracture diagnosis for statistical calculations to mirror the sonograph’s inability to exclude the presence of a fracture. Patients with missing data were excluded from calculations involving the missing data variable but were included in analyses not dependent on the missing data points.

## Results

**Figure 3:**
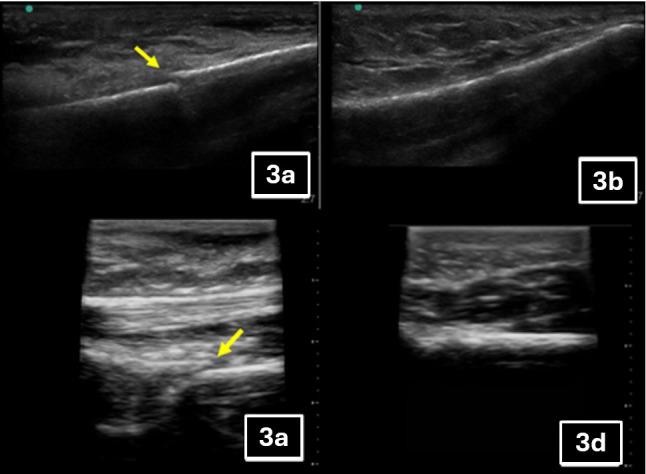
Fracture images. Sonosite M-Turbo images of fractured bone cortex (3a) and healthy cortex (3b). The arrow points to the fracture. Butterfly IQ+ (ophthalmic mode) images of fracture bone cortex (3c) and healthy cortex (3d). Arrows points to the fractures.

A total of 25 potential participants were considered for the study, three of whom met exclusion criteria. With this, 22 were eligible and included in the study, two of which had incomplete imaging. In the final analysis, 20 participants were included, as noted in Figure 1. There were four (20%) fractures identified, all of the distal fibula. Patient ages ranged from 15 to 59 years (average 23 years). There were four female patients in the study and 18 male patients (the study did allow for gender expansive identification; no patients identified as nonbinary). POCUS times ranged from 5 minutes to 30 minutes with a median length of 10 minutes (IQR: 9, 14.75). POCUS examiners performed between one and five scans in the study with an average of 1.7 scans preformed. Table 1 shows complete enrollment statistics. Of the scans performed, 17 participants received sonography using a Sonosite M-Turbo machine while three received Butterfly IQ+ sonography. Sonographic images of scans negative and positive for fracture are shown in Figure 3. Three of the fractures were observed with the Sonosite M-Turbo machine; one was observed with the Butterfly IQ+. There were no false negative scans. There was one false positive scan with an indeterminate scan on Butterfly IQ+ that ended up having no fracture. 

**Table 1:** Patient Demographics. This table describes the patients included in the study based on fracture versus no fracture. Each participant was allowed to contribute to more than one category if they multiple characteristics under the same area.

**Characteristics**	**Fracture (4)**	**No Fracture (16)**
**Sex **		
Male	2 (50%)	14 (87%)
Female	2 (50%)	2 (13%)
**Scan time**	10 9, 7	13 [9,15]^10^
**Age **		
Average age	39.3 [34, 50]	20.0 [16, 19]
Patients age 12-17 years	1 (25%)	9 (56%)
**OFAR Criteria **		
Lateral malleolus tenderness	4 (100%)	8 (50%)
Medial malleolus tenderness	0 (0%)	3 (19%)
5th Metatarsal tenderness	0 (0%)	5 (31%)
Navicular tenderness	0 (0%)	2 (13%)
Non ambulatory	1 (25%)	2 (13%)
**Method of injury **		
Inversion	2 (50%)	8 (%)
Eversion	1 (25%)	3 (19%)
Fall	2 (50%)	4 (25%)
Direct impact	0 (0%)	0 (0%)
Plantar hyper flexion	0 (0%)	1 (6%)
No method of injury data collected	1 (25%)	4 (25%)

Table 2 shows the diagnostic accuracy results of the scanning algorithm, the primary diagnosis (made by medical students), and the secondary diagnosis (made by POCUS experts). Overall, the diagnostic accuracy of novice POCUS practitioners was high, though with large confidence intervals. Bedside examiner SN and SP were as follows: SN 100% (95% CI: 40%-100%), SP 94% (95% CI: 70%-100%). Notably, the SN and SP were lower for the expert reviewers: SN 75% (95% CI: 19%- 99%) and SP 75% (95% CI: 48%, 93%). PPV for bedside examiner was 16.0 (95% CI: 2.40-106.7); for expert reviewers, PPV was 3.00 (95% CI: 3.00-8.32).

**Table 2:** Ottawa Foot and Ankle Rules Point of Care Ultrasound (OFAR-POCUS) algorithm diagnostic characteristics. Sensitivity (SN), specificity (SP), positive predictive value (PPV), negative predictive value (NPV), positive likelihood ratio (+LR), and negative likelihood ratio (-LR) results compared between the POCUS Examiners, who carried out the scan at bedside, with the retrospective expert reviewers.

	**SN**	**SP**	**PPV**	**NPV**	**+LR**	**-LR**
**POCUS Examiners**	100% (40%-100)	94% (70%-100%)	0.80 (0.28, 0.99)	1.00 (0.78, 1.00)	16.00(2.40,106.7)	0 (0.00, n/a)
**Expert Reviewers**	75% (19%-99%)	75% (48%-93%)	0.43 (0.10, 0.82)	0.92 (0.64, 1.00)	3.00 (1.08,8.32)	0.33(0.06,1.86)

## Discussion

This study found that novice POCUS practitioners were able to successfully utilize an OFAR-POCUS algorithm to identity fractures in a rural environment. In the study, examiners performed POCUS rapidly despite a short training format, and with high accuracy, though not statistically significant. 

Overall, the study found high SN. However, there was a large confidence interval likely due to the small sample size of the study. Notably there was a high +LR, suggesting that POCUS may be beneficial in identifying fractures. In the study, POCUS was rapidly performed; the median POCUS exam length was 10 minutes, indicating pragmatic clinical feasibility. This contrasts with needing patients to be transferred to another facility to have radiographs performed and then read, which could take hours or longer. Moreover, POCUS examiners performed an average of 1.7 scans in the study, so it is reasonable to postulate this number would decrease with more clinical repetitions. A well, there were no observed fractures over areas without point tenderness, and the algorithm had five scanning views (medial malleolus, lateral malleolus, inferior view of 5^th^ metatarsal, lateral view of 5^th^ metatarsal, and superior view of 5^th^ metatarsal). Therefore, using a modified technique of only scanning over areas of tenderness would likely reduce the scan times and without sacrificing SN or NPV. Additionally, this study’s 10-minute median sonographic exam length was achieved after an average of 1.7 repetitions; further repetitions would likely also reduce scan time.

Previous investigations with adult patients have demonstrated positive likelihood ratios of 10-83 with POCUS fracture algorithms 5, 6, in accord with this study’s +LR= 16 (95% Cl, 2.15, 48) for POCUS examiners. Moreover, the high bedside OFAR-POCUS SN and SP was in line with previous adult OFAR-POCUS studies which yielded SN=80%-94.9% and SP=99.2%-100% 5, 6. 

There are fewer investigations with pediatric populations, although one study demonstrates +LR of 3.1 (95% CI, 1.27–7.54) 11. Another study showed SN=56% (95% CI, 23%-85%) and SP=82% (95% CI, 66%-92%) when screening for foot and ankle fractures in pediatric patients aged 0-21 years using ultrasound 10. The POCUS examiner SN, SP, and +LR findings from this study (SN=100% (95% Cl, 40%-100%), SP=94% (95% Cl, 70-100%), and +LR=16.00 (2.40, 106.7)), overlap with the reported adult and pediatric published values from the aforementioned experiments. This consensus of data could suggest a role in OFAR-POCUS in ruling out ankle fractures, but larger multicenter studies would need to verify this possibility.

Accordingly, these findings imply that a combination of history and physical with POCUS, as was used in this study with the novice bedside examiners, has the highest diagnostic accuracy which highlights the strengths of POCUS as a bedside test. Indeed, no fractures were observed in areas that were not tender to palpation. Therefore, possible future protocols could limit sonography to regions where patients have pain (usually only one or two of the Ottawa sites) which would likely reduce exam time even further. Furthermore, it was feasible for novice learners to use a diagnostic algorithm including POCUS to identify fracture SN and SP. To our knowledge, this is the first study evaluating a diagnostic algorithm for patients presenting with foot and ankle injuries using foot and ankle POCUS in a rural population and one of the few evaluating it in an adolescent patient population.

## Limitations

This study has several limitations. Perhaps the greatest limitation is its small sample size. We enrolled 22 patients who had a total of four fractures, all of which were of the distal fibula. As this study had low patient enrollment, the confidence intervals on all statistics are wide. This limitation makes drawing firm conclusions on accuracy difficult. We further are unable to draw conclusions on accuracy of diagnosing foot fractures specifically, given the lack of foot fractures in our population. We also included indeterminate findings as positive results which likely biased our results. We did this as the aim of OFAR-POCUS is to rule out fractures and an indeterminate result would still reflect the inability to rule out a fracture. Still, we were able to demonstrate it was feasible to train novices to perform POCUS of the foot and ankle after a relatively brief training session, and additional studies with larger sample sizes are needed to further assess accuracy.

The expert reviewers explicitly detailed how being blinded from patient age made identifying growth plates versus possible fractures difficult. Moreover, they did not know which scans corresponded to where patients had pain, while the novice sonographers did. Accordingly, when the bedside examiner saw sonographic features that looked similar to fractures but were in adolescent patients and at locations with no pain, they had low suspicion while reviewers could only rely on the image. This likely gave the POCUS examiners a significant advantage in distinguishing probable fractures from growth plates, normal growing bone, and sonographic artifacts. Realistic clinical practice would resemble the bedside examiners’ experience as real-world examiners would not be blinded from the patient they are examining.

 Furthermore, this study was a single center study relying on a single instructor, which makes the findings difficult to generalize to other settings. We also utilized a convenience sample of patients which could introduce bias in patient selection. 

## Conclusions

We successfully implemented an algorithm including POCUS for identification of foot and ankle fractures in adolescent and adult patients in a rural setting. Novice POCUS examiners were able to quickly learn and use an OFAR-POCUS algorithm in a clinical setting. Larger, multicenter studies are needed to further investigate its accuracy.

## Disclosure Statement 

The authors declare that they have no competing interests.

## Supplementary Material

**Supporting Figure 1:**